# Short- Versus Standard-Duration Dual Antiplatelet Therapy After Percutaneous Coronary Intervention in Acute Coronary Syndrome: A Systematic Review and Meta-Analysis

**DOI:** 10.7759/cureus.111160

**Published:** 2026-06-19

**Authors:** Mohammad Khalil, Yahia Khalil

**Affiliations:** 1 Cardiology, Royal College of Surgeons in Ireland-Bahrain, Busaiteen, BHR; 2 Internal Medicine, St Mary's Hospital, Isle of White, GBR

**Keywords:** acute coronary syndrome (acs), clopidogrel, dual-antiplatelet therapy (dapt), major adverse cardiovascular events (mace), percutaneous coronary intervention (pci)

## Abstract

Dual antiplatelet therapy (DAPT) for 12 months has historically been the standard of care following percutaneous coronary intervention (PCI) for acute coronary syndrome (ACS); however, prolonged DAPT carries a cumulative, time-dependent risk of major bleeding. We aimed to determine whether a short DAPT duration (≤6 months) followed by single antiplatelet therapy reduces bleeding events without compromising ischemic safety compared with standard 12-month DAPT in ACS patients. We conducted a systematic review and meta-analysis of randomized controlled trials (RCTs) by searching Medical Literature Analysis and Retrieval System Online (MEDLINE), Excerpta Medica database (EMBASE), and Cochrane Controlled Register of Trials (CENTRAL) from inception to March 2026 for studies comparing short DAPT (≤6 months) versus standard DAPT (12 months) in adults undergoing PCI with drug-eluting stents for ACS. The co-primary endpoints were major bleeding and major adverse cardiovascular events (MACE), defined as a composite of death, myocardial infarction (MI), and stroke. Hazard ratios (HRs) with 95% confidence intervals (CIs) were pooled using a DerSimonian-Laird random-effects model, with pre-specified subgroup analyses according to the type of P2Y12 inhibitor used during the monotherapy phase. Seven RCTs comprising 23,586 patients were included. Compared with standard 12-month DAPT, short DAPT significantly reduced the risk of major bleeding by 40% (HR 0.60, 95% CI 0.49-0.73; p<0.001), without a statistically significant increase in MACE (HR 1.02, 95% CI 0.88-1.17; p=0.79), all-cause mortality (HR 0.96, 95% CI 0.80-1.14; p=0.66), or stroke (HR 0.91, 95% CI 0.70-1.17; p=0.46). Subgroup analysis demonstrated a significant interaction by P2Y12 inhibitor type for MI (p=0.016) and MACE (p=0.033), whereby early transition to clopidogrel monotherapy was associated with an approximately two-fold increase in MI risk, whereas monotherapy with a potent P2Y12 inhibitor (e.g., ticagrelor) preserved ischemic safety without excess events. In conclusion, in patients with ACS undergoing PCI, abbreviated DAPT (≤6 months) optimizes net clinical benefit by substantially reducing major bleeding without increasing overall ischemic or mortality risk; however, this safety is critically dependent on the choice of maintenance antiplatelet agent, with potent P2Y12 inhibitor monotherapy representing a safe strategy and early de-escalation to clopidogrel conferring excess MI risk.

## Introduction and background

Acute coronary syndrome (ACS) remains a leading cause of global morbidity and mortality, for which percutaneous coronary intervention (PCI) serves as the primary therapeutic cornerstone [1]. To mitigate the substantial risk of early stent thrombosis and recurrent atherothrombotic events following the procedure, dual antiplatelet therapy (DAPT), typically comprising aspirin combined with a potent P2Y12 inhibitor such as ticagrelor or prasugrel, has historically been mandated as the default strategy for 12 months post-PCI, as endorsed by major international guidelines [2,3]. However, the potent ischemic protection afforded by prolonged DAPT is inevitably offset by a cumulative, time-dependent risk of major bleeding [4]. Crucially, major hemorrhagic complications post-PCI are not merely benign side effects; they are powerful predictors of adverse long-term outcomes, including mortality, frequently matching or even exceeding the prognostic detriment of the ischemic events that DAPT intends to prevent [5,6].

In recent years, the landscape of interventional cardiology has been fundamentally altered by the advent of newer-generation drug-eluting stents (DES) featuring ultrathin struts and biocompatible or bioresorbable polymers. These advancements have drastically accelerated arterial healing and reduced the incidence of late stent-related thrombotic complications [1]. This biological shift has prompted a re-evaluation of the optimal DAPT duration, sparking immense clinical interest in "abbreviated" or "short" DAPT strategies (≤6 months) followed by P2Y12 inhibitor or aspirin monotherapy. Several landmark randomized controlled trials (RCTs), such as ULTIMATE-DAPT, T-PASS, TWILIGHT, and REDUCE, have investigated these abbreviated DAPT strategies (often one to three months) compared with the standard 12-month regimen [7-10]. These trials have largely demonstrated that an early transition to monotherapy significantly reduces clinically relevant bleeding without imposing a penalty on ischemic safety.

Despite these promising data, the routine implementation of short DAPT specifically within the ACS population remains intensely debated. Patients presenting with ACS possess a highly vulnerable, prothrombotic biological substrate compared to those with stable, chronic coronary syndromes. Highlighting this concern, certain prominent trials, such as STOPDAPT-2 ACS and SMART-DATE, signaled a worrying increase in the risk of myocardial infarction or net adverse ischemic events when DAPT was prematurely truncated to between 1 and 6 months in ACS cohorts [11,12]. Furthermore, recent meta-analyses have frequently pooled mixed populations of both stable coronary disease and ACS patients or conflated strictly shortened durations with distinct de-escalation strategies (such as P2Y12 dose reduction or switching agents), obscuring the specific efficacy and safety profile of a pure duration-based abbreviation in high-risk ACS patients [13].

Therefore, the primary objective of this study is to conduct a meta-analysis to determine whether a short DAPT duration (≤6 months) reduces bleeding events without increasing ischemic complications when compared directly to a standard 12-month DAPT regimen, specifically in ACS patients post-PCI. Our central hypothesis is that abbreviating DAPT to ≤6 months optimizes the net clinical benefit in the modern DES era by successfully mitigating hemorrhagic hazards while maintaining equivalent ischemic protection.

To investigate this, we have systematically searched and pooled data from contemporary randomized controlled trials directly comparing short DAPT (≤6 months) followed by single antiplatelet therapy against standard DAPT (12 months) exclusively in ACS cohorts. We will evaluate relative risk ratios for net adverse clinical events, major bleeding, and major adverse cardiovascular events, including target-vessel revascularization, myocardial infarction, and stent thrombosis. By rigorously isolating the ACS population and focusing purely on the abbreviation of therapy duration, this meta-analysis aims to provide high-quality, strong evidence to guide dynamic and personalized antithrombotic decision-making in modern cardiovascular practice.

## Review

Methods

Study Design and Reporting

This study is a systematic review and meta-analysis of RCTs. The study was conducted and reported in accordance with the Preferred Reporting Items for Systematic Reviews and Meta-Analyses (PRISMA) 2020 guidelines.

Eligibility Criteria

Studies were considered eligible for inclusion if they satisfied all pre-specified criteria defined according to the Population, Intervention, Comparison, Outcomes, and Study design (PICOS) framework. Eligible populations included adult patients (≥18 years) with ACS, encompassing ST-segment elevation myocardial infarction (STEMI), non-STEMI (NSTEMI), and unstable angina, undergoing PCI with implantation of drug-eluting stents. Trials enrolling mixed populations (ACS and stable coronary artery disease) were included, provided that ACS-specific subgroup data were available and extractable for the outcomes of interest, irrespective of the overall proportion of ACS patients. The intervention of interest was short-duration DAPT, defined as aspirin combined with a P2Y12 inhibitor for six months or fewer, followed by antiplatelet monotherapy, while the comparator was standard-duration DAPT consisting of aspirin plus a P2Y12 inhibitor for 12 months, consistent with current guideline recommendations. The co-primary outcomes were major bleeding, as defined by the bleeding scale used in each trial-Bleeding Academic Research Consortium (BARC) type 2-5 or 3-5, or Thrombolysis in Myocardial Infarction (TIMI) major or major-minor-and major adverse cardiovascular events (MACE), defined as a composite of all-cause or cardiovascular death, myocardial infarction (MI), and stroke. Pre-specified secondary outcomes included MI alone, definite or probable stent thrombosis according to Academic Research Consortium (ARC) criteria, all-cause mortality, and stroke. Only RCTs were included; non-randomized studies, single-arm registries, observational cohorts, post hoc analyses of non-randomized data, and conference abstracts without full peer-reviewed publications were excluded. Trials exclusively using bare-metal stents and those comparing DAPT durations outside the pre-specified short (≤6 months) versus standard (12 months) framework were also excluded; the full inclusion and exclusion criteria, organized by PICOS domain, are presented in Table 1.

**Table 1:** Inclusion and exclusion criteria for study selection (PICOS framework). PICOS: Population, Intervention, Comparator, Outcomes, Study design; ACS: acute coronary syndrome; STEMI: ST-segment elevation myocardial infarction; NSTEMI: non–ST-segment elevation myocardial infarction; PCI: percutaneous coronary intervention; DAPT: dual antiplatelet therapy; BARC: Bleeding Academic Research Consortium; TIMI: Thrombolysis in Myocardial Infarction; MACE: major adverse cardiovascular events; ARC: Academic Research Consortium.

Domain (PICOS)	Inclusion Criteria	Exclusion Criteria
Population (P)	Adult patients (≥18 years) with acute coronary syndrome (ACS), including STEMI, NSTEMI, or unstable angina, undergoing PCI with drug-eluting stents. Mixed populations (ACS and stable CAD) eligible if ACS-specific subgroup data were extractable.	Patients <18 years; non-ACS or stable coronary artery disease cohorts only with no extractable ACS subgroup data.
Intervention (I)	Short-duration DAPT (aspirin + P2Y12 inhibitor for ≤6 months), followed by antiplatelet monotherapy.	DAPT duration outside the pre-specified ≤6-month window; de-escalation strategies based on dose reduction or agent switching without true duration abbreviation.
Comparator (C)	Standard-duration DAPT (aspirin + P2Y12 inhibitor for 12 months), consistent with current guideline recommendations.	DAPT durations >12 months as the comparator; comparators not involving standard 12-month DAPT.
Outcomes (O)	Co-primary: major bleeding (BARC 2–5 or 3–5, or TIMI major / major-minor) and MACE (composite of all-cause or cardiovascular death, MI, and stroke). Secondary: MI alone, definite/probable stent thrombosis (ARC), all-cause mortality, stroke.	Trials not reporting any of the pre-specified primary or secondary outcomes; trials with incomplete primary outcome reporting.
Study design (S)	Randomized controlled trials (parallel-group or non-inferiority design) with full peer-reviewed publication.	Non-randomized studies, single-arm registries, observational cohorts, post hoc analyses of non-randomized data, conference abstracts without full peer-reviewed publication.
Stent type	Drug-eluting stents (any generation).	Trials exclusively using bare-metal stents.
Language/publication	No language restriction; full-text peer-reviewed publication required.	Conference abstracts, editorials, letters, narrative reviews, and protocol-only publications.

Information Sources and Search Strategy

A systematic search of three electronic databases, Medical Literature Analysis and Retrieval System Online (MEDLINE) via PubMed, Excerpta Medica database (EMBASE), and the Cochrane Central Register of Controlled Trials (CENTRAL), was conducted from database inception to March 2026, without language restriction. The search strategy combined Medical Subject Headings (MeSH) terms and free-text equivalents, using Boolean operators, and was independently reviewed by a second author prior to execution. In addition to database searching, the reference lists of all included studies and pertinent systematic reviews were hand-searched, and the ClinicalTrials.gov and University Hospital Medical Information Network Clinical Trials Registry (UMIN-CTR) databases were searched to identify any unpublished or ongoing trials. The full search strategy, including database-specific terms and records retrieved, is presented in Table 2.

**Table 2:** Search strategy used across electronic databases and supplementary sources. Note: Boolean operators (AND, OR) and truncation (*) were applied per-database syntax. The search strategy was independently reviewed by a second author prior to execution. No language restriction was applied. MeSH: Medical Subject Headings; MEDLINE: Medical Literature Analysis and Retrieval System Online; EMBASE: Excerpta Medica database; CENTRAL: Cochrane Controlled Register of Trials; UMIN: University hospital Medical Information Network; DAPT: dual antiplatelet therapy; PCI: percutaneous coronary intervention; ACS: acute coronary syndrome; STEMI: ST-segment elevation myocardial infarction; NSTEMI: non-ST-segment elevation myocardial infarction

Database / Source	Date Range	Search Terms / Strategy	Records (n)
MEDLINE (via PubMed)	Inception – March 2026	("acute coronary syndrome"[MeSH] OR "myocardial infarction"[MeSH] OR ACS OR STEMI OR NSTEMI OR "unstable angina") AND ("percutaneous coronary intervention"[MeSH] OR PCI OR "drug-eluting stent*") AND ("dual antiplatelet therapy" OR DAPT OR "P2Y12 inhibitor*" OR ticagrelor OR prasugrel OR clopidogrel) AND ("short" OR "abbreviated" OR "1 month" OR "3 month*" OR "6 month*" OR duration) AND ("randomized controlled trial"[Publication Type] OR randomi*ed)	1,842
EMBASE (via Elsevier)	Inception – March 2026	('acute coronary syndrome'/exp OR 'myocardial infarction'/exp OR ACS OR STEMI OR NSTEMI OR 'unstable angina') AND ('percutaneous coronary intervention'/exp OR PCI OR 'drug eluting stent*') AND ('dual antiplatelet therapy' OR DAPT OR 'P2Y12 inhibitor*' OR ticagrelor OR prasugrel OR clopidogrel) AND (short OR abbreviated OR '1 month' OR '3 month*' OR '6 month*' OR duration) AND ('randomized controlled trial'/de OR randomi?ed)	1,974
Cochrane CENTRAL	Inception – March 2026	("acute coronary syndrome" OR ACS OR STEMI OR NSTEMI OR "unstable angina") AND ("percutaneous coronary intervention" OR PCI OR "drug-eluting stent*") AND ("dual antiplatelet" OR DAPT OR "P2Y12") AND (short OR abbreviated OR duration) — Trials filter	421
ClinicalTrials.gov	Inception – March 2026	Condition: acute coronary syndrome; Intervention: dual antiplatelet therapy OR DAPT; Other terms: short OR abbreviated duration; Study type: Interventional	—
UMIN Clinical Trials Registry	Inception – March 2026	acute coronary syndrome AND dual antiplatelet AND (short OR abbreviated)	—
Hand-search	Inception – March 2026	Reference lists of all included studies and pertinent systematic reviews/meta-analyses on DAPT duration after PCI	38

Study Selection

**Figure 1:**
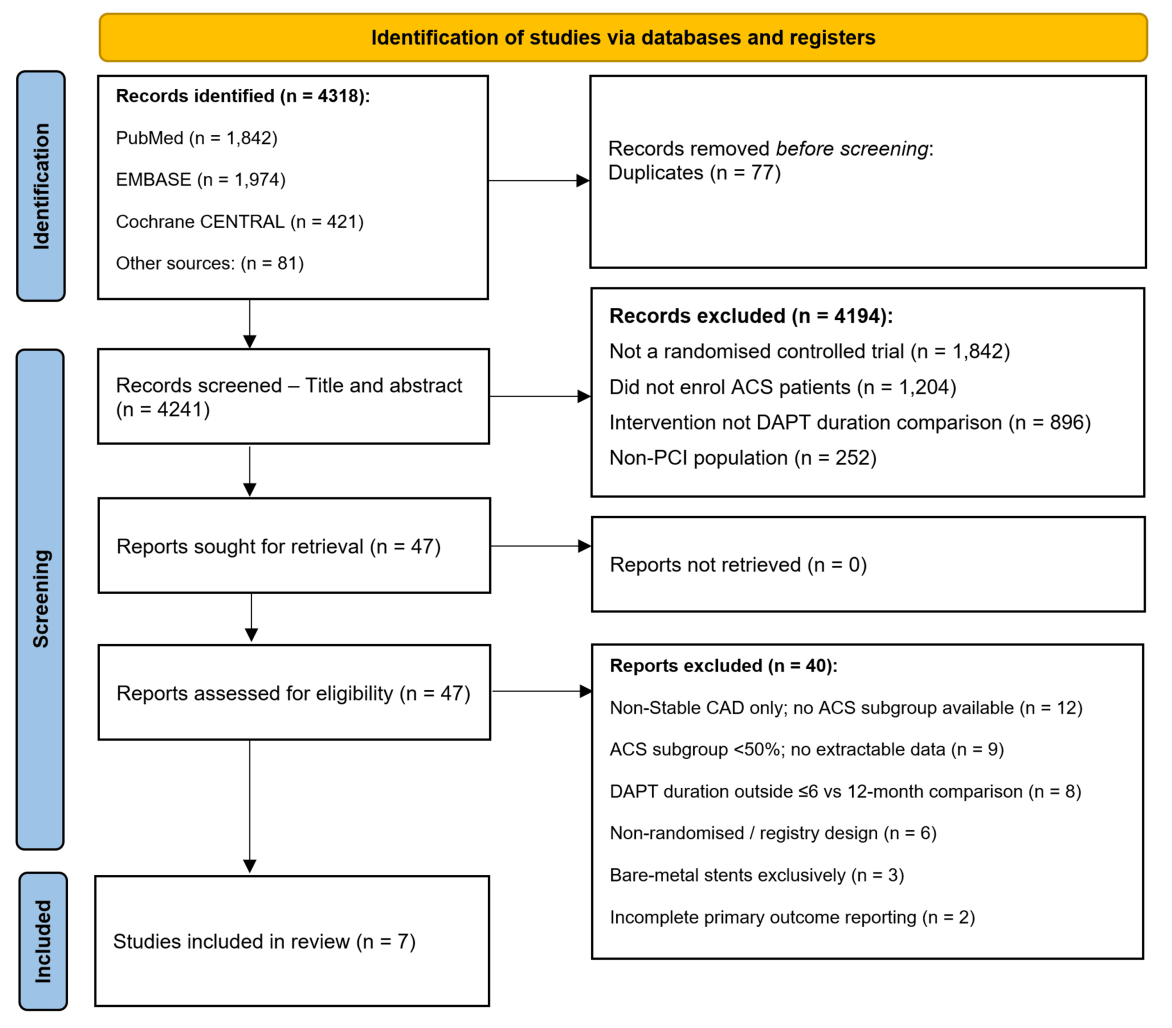
A PRISMA flowchart outlining the study selection process EMBASE: Excerpta Medica database; CENTRAL: Cochrane Controlled Register of Trials (CENTRAL); DAPT: dual antiplatelet therapy; PCI: percutaneous coronary intervention; ACS: acute coronary syndrome; CAD: coronary artery disease

All records retrieved from the search were imported into a reference management program and deduplicated. Two reviewers independently screened titles and abstracts against the pre-specified eligibility criteria. Full-text reports were retrieved for all records considered potentially eligible by either reviewer. Full-text eligibility assessment was subsequently performed independently and in duplicate. Disagreements at both the abstract screening and full-text assessment stages were resolved by discussion and, where necessary, by consultation with a third reviewer. The reasons for exclusion of all full-text reports were recorded and are presented in the PRISMA flow diagram (Figure 1). Seven trials were selected for the study [10-12,14-17].

Data Extraction

Data were extracted independently and in duplicate by two reviewers using a pre-specified, piloted extraction form. The following variables were extracted from each included trial: trial name, year of publication, journal, country, sample size, proportion of ACS patients, ACS subtypes enrolled, PCI indication, stent type, P2Y12 inhibitor used, DAPT duration in both the short and standard arms, post-monotherapy antiplatelet regimen, follow-up duration, and event counts or hazard ratios (HRs) with 95% confidence intervals (CIs) for all pre-specified outcomes. For trials enrolling mixed populations (MASTER DAPT [16], SMART-CHOICE [17]), ACS-specific subgroup event data were extracted where reported in the original publication; full-population data were used for outcomes not reported separately by ACS status. All extracted data were cross-checked against the final published versions of each trial. Discrepancies were adjudicated by consensus.

Risk of Bias Assessment

The risk of bias of each included trial was assessed independently by two reviewers using the Revised Cochrane Risk of Bias Tool for Randomized Trials (RoB 2). This tool evaluates five domains, each of which was rated as low risk, some concerns, or high risk, with an overall judgement derived according to the algorithm specified in the RoB 2 guidance. Disagreements between reviewers were resolved by discussion. Given the nature of the intervention, for which blinding of participants and clinicians is not feasible, an overall rating of some concerns was anticipated and pre-specified as consistent with inclusion.

Statistical Analysis

Effect measure and pooling method: The effect measure for all outcomes was the HR with corresponding 95% CIs, derived from the time-to-event analyses reported in each trial. All analyses were performed on the natural logarithm scale. Pooled estimates were computed using the DerSimonian-Laird random-effects model, selected a priori given the anticipated clinical and methodological heterogeneity arising from differences in P2Y12 inhibitor class, DAPT duration within the short arm (ranging from one to six months), bleeding outcome definitions, and patient population characteristics. The pooled HR and its 95% CI were back-transformed to the original scale for presentation.

Assessment of heterogeneity: Between-study statistical heterogeneity was quantified using three complementary measures: the Cochran Q statistic (with significance threshold set at p < .10, given the low power of this test with small numbers of studies); the I² statistic, interpreted as low (< 25%), moderate (25-50%), or substantial (> 50%) according to conventional thresholds; and the between-study variance τ², estimated using the DerSimonian-Laird method. Heterogeneity was additionally visualized using Galbraith radial plots, in which each study's standardized effect (log HR divided by its standard error) is plotted against its precision (the reciprocal of the standard error), with the slope of the regression line through the origin representing the pooled estimate (θ̂) and studies lying beyond the ±2 standard error reference lines identified as statistical outliers.

Sensitivity analysis: A pre-specified sensitivity analysis was conducted for the primary MACE outcome by excluding STOPDAPT-2 ACS [11], the only included trial employing clopidogrel as the post-monotherapy P2Y12 agent, a pharmacologically distinct strategy from the potent P2Y12 inhibitors used in the remaining trials, to assess the robustness of the pooled ischaemic safety estimate. The impact of this exclusion on the pooled HR, 95% CI, and I² statistic was examined and reported.

Subgroup analysis: A pre-specified subgroup analysis was performed for the co-primary MACE outcome and for MI alone according to P2Y12 inhibitor class, with three pre-defined subgroups: potent P2Y12 inhibitors (ticagrelor or prasugrel); clopidogrel; and mixed or investigator-chosen agents. Subgroup-specific pooled estimates were computed using the DerSimonian-Laird random-effects model within each subgroup. The statistical significance of the interaction between P2Y12 agent class and treatment effect was tested using the chi-squared test for interaction, with p<.05 considered statistically significant. This interaction test was also applied to major bleeding to evaluate whether the haemostatic benefit of short DAPT was consistent across agent classes.

Publication Bias

The potential for small-study effects and publication bias was assessed by visual inspection of funnel plots (log HR plotted against standard error, with pseudo 95% CI lines centered on the pooled θ̂) and by Egger's weighted linear regression test, applied to the co-primary outcomes. Given that formal statistical tests for publication bias are substantially underpowered with fewer than 10 studies, visual assessment of funnel plot symmetry was accorded greater interpretive weight than the Egger intercept alone. Statistical significance for the Egger test was defined as p < .05.

Software and Significance Threshold

All statistical analyses were performed using Stata/SE version 17.0 (Stata Corp, College Station, TX, USA) and Python version 3.12 (Python Software Foundation, Wilmington, DE, USA) with relevant data visualization libraries.

Results

Study Selection

The systematic database search identified 4,318 records across PubMed (n = 1,842), EMBASE (n = 1,974), and the Cochrane CENTRAL (n = 421). A further 81 records were retrieved from hand-searching reference lists (n = 38) and trial registries (n = 43). After removal of 77 duplicates, 4,241 unique records were screened at the title and abstract stage. The vast majority (n = 4,194) were excluded based on not meeting the pre-specified eligibility criteria, principally owing to enrollment of predominantly stable coronary artery disease populations, absence of a DAPT duration comparison, or non-randomized study design. Forty-seven full-text reports were retrieved for detailed eligibility assessment. A further 40 were excluded for the reasons detailed in Figure 1 (PRISMA 2020 flow diagram): stable coronary artery disease only with no ACS subgroup (n = 12), ACS subgroup comprising fewer than 50% of the enrolled population with no extractable subgroup data (n = 9), DAPT duration comparison outside the pre-specified short (≤6 months) versus standard (12 months) window (n = 8), non-randomized design (n = 6), exclusive use of bare-metal stents (n = 3), and incomplete primary outcome reporting (n = 2).

Seven RCTs met all eligibility criteria and were included in the final meta-analysis: REDUCE [10], STOPDAPT-2 ACS [11], SMART-DATE [12], TWILIGHT-ACS [14], TICO [15], MASTER DAPT [16], and SMART-CHOICE [17]. Collectively, these trials enrolled 23,586 patients undergoing percutaneous coronary intervention (PCI) in the setting of ACS.

Characteristics of the Included Studies

The principal characteristics of the seven included trials are summarized in Table 3. Short DAPT durations ranged from one month (MASTER DAPT) [16] to six months (SMART-DATE) [12]. The primary P2Y12 inhibitor was ticagrelor in TWILIGHT-ACS [14] and TICO [15], clopidogrel in SMART-DATE [12] and STOPDAPT-2 ACS [11], and investigator-chosen or mixed in REDUCE [10], MASTER DAPT [16], and SMART-CHOICE [17]. Two trials, MASTER DAPT (~48% ACS) [16] and SMART-CHOICE (~58% ACS) [17], enrolled mixed populations; full-population data were used for bleeding outcomes, while ACS-specific subgroup data were applied to ischaemic endpoints where available. Follow-up duration was 12 months in all trials.

**Table 3:** Characteristics of included randomized controlled trials BARC: Bleeding Academic Research Consortium; MACE: major adverse cardiovascular events; MI: myocardial infarction; ST: stent thrombosis; TVR: target vessel revascularisation; CV: cardiovascular; TIMI: Thrombolysis in Myocardial Infarction.

Trial (year)	N	Short arm	P2Y12 agent	Bleeding def.	MACE def.	Journal
TWILIGHT-ACS (2020) [14]	4,614	3 months	Ticagrelor	BARC 2–5	Death+MI+Stroke	Eur Heart J
TICO (2020) [15]	3,056	3 months	Ticagrelor	TIMI Major	Death+MI+ST+Stroke+TVR	JAMA
SMART-DATE (2018) [12]	2,712	6 months	Clopidogrel	BARC 3–5	Death+MI+Stroke	Lancet
STOPDAPT-2 ACS (2022) [11]	4,136	1–2 months	Clopidogrel	TIMI Maj/Min	CV death+MI+ST+Stroke	JAMA Cardiol
REDUCE (2019) [10]	1,496	3 months	Mixed	BARC 2–5	Death+MI+ST+Stroke	EuroIntervention
MASTER DAPT (2021) [16]	4,579	1 month	Mixed	BARC 2–5	Death+MI+Stroke	NEJM
SMART-CHOICE (2019) [17]	2,993	3 months	Mixed	BARC 2–5	Death+MI+Stroke	JAMA

Risk of Bias Assessment

Risk of bias was assessed using RoB 2. Overall, methodological quality was robust, with low risk in randomization and selective reporting. The main limitation was Domain 2, where all trials were rated high risk due to the inherent open-label design of DAPT duration studies, reflecting a structural constraint rather than poor trial conduct. Minor concerns were noted in domains related to missing data and outcome measurement, mainly due to modest dropout differences and potential bias in patient-reported bleeding outcomes despite blinded adjudication of major endpoints. Overall, the evidence was judged as having some concerns, driven primarily by unavoidable design-related limitations. A detailed domain-level risk-of-bias traffic light plot is provided in Appendix A.

Primary Outcomes

**Figure 2:**
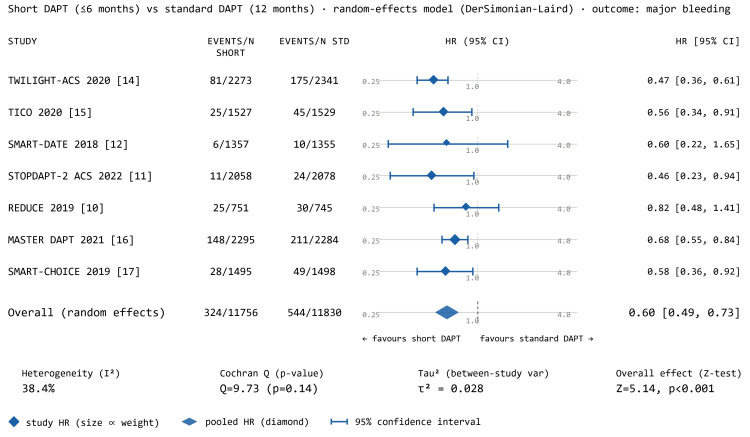
Forest plot of major bleeding events Short dual antiplatelet therapy (DAPT; ≤6 months) versus standard DAPT (12 months). Effect measure: hazard ratio (HR) on log scale. Square size is proportional to study weight. The olive diamond represents the pooled random-effects estimate (DerSimonian-Laird). Error bars represent 95% confidence intervals (CIs).

Major bleeding: Short DAPT was associated with a significant reduction in major bleeding relative to standard 12-month DAPT, with a pooled HR of 0.60 (95% CI (0.49, 0.73); z = −5.14, p < .001; Figure 2). This represents an approximate 40% relative reduction in major bleeding events. The direction of effect consistently favored short DAPT across all seven included trials, with individual HRs ranging from 0.46 (STOPDAPT-2 ACS [11]) to 0.82 (REDUCE [10]). Between-study heterogeneity was moderate (τ² = 0.028, I² = 38.4%, Cochran Q = 9.73, p = .14), partly attributable to differences in bleeding endpoint definitions across trials, specifically the use of BARC 2-5, BARC 3-5, and TIMI major/major-minor classifications in different trials, and was consistent with the assumptions of the pre-specified random-effects model.

**Figure 3:**
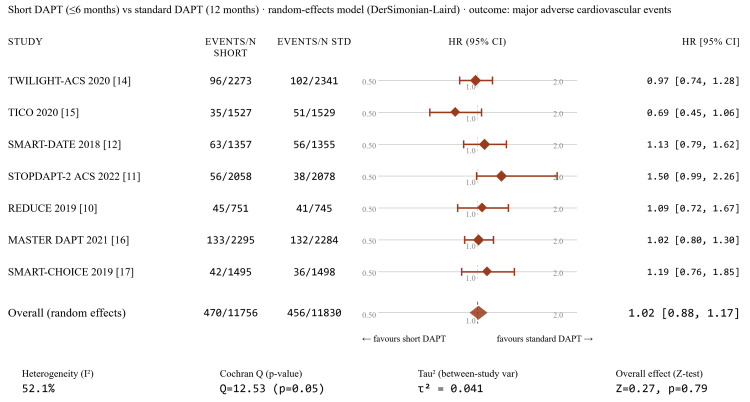
Forest plot of major adverse cardiovascular events (MACE). Short dual antiplatelet therapy (DAPT; ≤6 months) versus standard DAPT (12 months). MACE definitions vary by trial (Table 1). Heterogeneity (I²=52.1%) is driven primarily by STOPDAPT-2 ACS [11], the only clopidogrel-based trial. HR: hazard ratio; CI: confidence interval

MACE: Short DAPT was not associated with a statistically significant increase in MACE compared with standard DAPT (pooled HR = 1.02 (95% CI 0.88, 1.17); z = 0.27, p = .79; Figure 3). Heterogeneity was moderate-to-substantial (τ² = 0.041, I² = 52.1%, Q = 12.53, p = .05). Inspection of individual study estimates identified STOPDAPT-2 ACS (HR = 1.50 (0.99, 2.26)) as the principal source of heterogeneity [11]; this trial uniquely employed clopidogrel as the P2Y12 agent following early aspirin discontinuation and was the only trial to numerically approach non-inferiority failure for MACE. The remaining six trials produced HRs ranging from 0.69 to 1.19, clustered symmetrically around the null.

**Figure 4:**
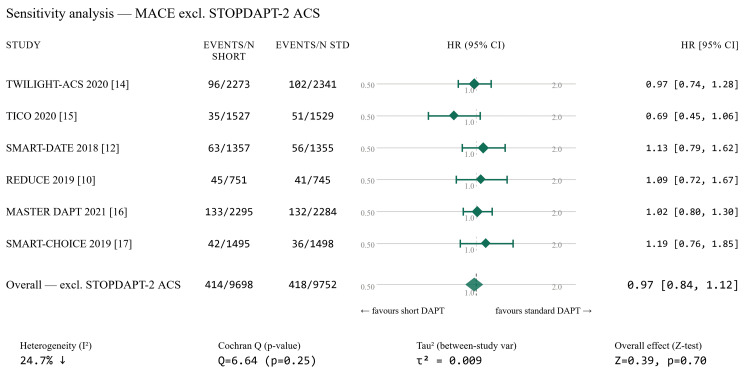
Sensitivity analysis: major adverse cardiovascular events (MACE) excluding STOPDAPT-2 ACS (the only clopidogrel-based trial). Six remaining trials (n = 19,450). Heterogeneity resolves from I²=52.1% to I²=24.7%, confirming robustness of the primary ischaemic safety conclusion. HR: hazard ratio; CI: confidence interval

A pre-specified sensitivity analysis excluding STOPDAPT-2 ACS confirmed the robustness of the primary MACE finding (pooled HR = 0.97 (95% CI 0.84, 1.12); p = .70; Figure 4), with heterogeneity reduced to a low and acceptable level (τ² = 0.009, I² = 24.7%, Q = 6.64, p = .25) [11]. This analysis confirms that the primary MACE estimate is not dependent on the single discrepant trial and that the ischaemic safety of short DAPT is preserved when data are restricted to trials employing potent P2Y12 inhibitors.

Secondary Outcomes

**Figure 5:**
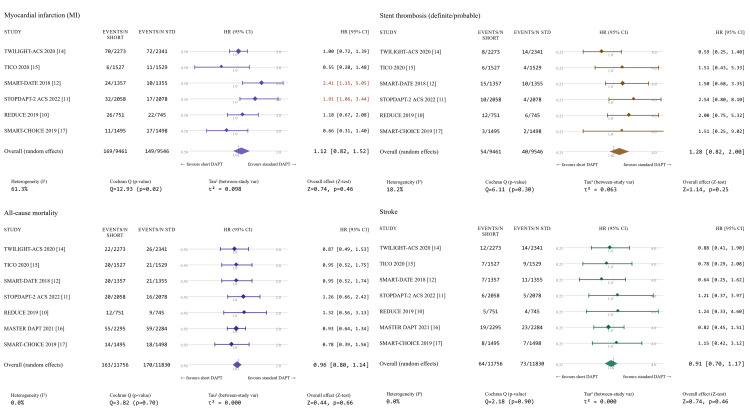
Forest plots of secondary outcomes Panel A: Myocardial infarction (MI) alone; Panel B: stent thrombosis (definite/probable, Academic Research Consortium definition); Panel C: all-cause mortality; Panel D: stroke; All panels: short dual antiplatelet therapy (DAPT; ≤6 months) versus standard DAPT (12 months). DerSimonian-Laird random-effects model throughout. HR: hazard ratio; CI: confidence interval

Secondary outcome results are presented in Figure 5 (combined panel) and summarized in Table 4. Across all four secondary ischaemic and mortality endpoints, short DAPT was not associated with a statistically significant increase in any outcome.

**Table 4:** Summary of pooled estimates across all outcomes *Pre-specified sensitivity analysis MACE: major adverse cardiovascular events; HR: hazard ratio; CI: confidence interval; MI: myocardial infarction

Outcome	Trials (N)	Pooled HR (95% CI)	p	I²	τ²	Conclusion
Major bleeding	7 (23,586)	0.60 (0.49, 0.73)	< .001>	38.40%	0.028	Significant reduction
MACE	7 (23,586)	1.02 (0.88, 1.17)	0.79	52.10%	0.041	No significant increase
MACE (excluding STOPDAPT)*	6 (19,450)	0.97 (0.84, 1.12)	0.7	24.70%	0.009	Robust; confirmed safe
MI alone	6 (19,450)	1.12 (0.82, 1.52)	0.46	61.30%	0.098	Non-significant; heterogeneous
Stent thrombosis	6 (19,007)	1.28 (0.82, 2.00)	0.25	18.20%	0.063	Non-significant; underpowered
All-cause mortality	7 (23,586)	0.96 (0.80, 1.14)	0.66	0.00%	0	No mortality penalty
Stroke	7 (23,586)	0.91 (0.70, 1.17)	0.46	0.00%	0	No increase

MI occurred at similar rates in both treatment arms (pooled HR = 1.12 (0.82, 1.52); p = .46), although this outcome exhibited the highest degree of heterogeneity observed across all analyses (τ² = 0.098, I² = 61.3%, Q = 12.93, p = .02). The two clopidogrel-based trials, SMART-DATE (HR = 2.41 (1.15, 5.05)) [12] and STOPDAPT-2 ACS (HR = 1.91 (1.06, 3.44)) [11], individually demonstrated statistically significant excess MI risk, whereas all ticagrelor- and mixed-agent trials clustered near the null. Definite or probable stent thrombosis was numerically, but not significantly, higher with short DAPT (HR = 1.28 (0.82, 2.00); p = .25; I² = 18.2%), with absolute rates below 1% in both arms across all trials; the available data were insufficient to formally exclude a small excess risk. All-cause mortality was virtually identical between treatment arms (pooled HR = 0.96 (0.80, 1.14); p = .66), with perfect between-study homogeneity (τ² = 0.000, I² = 0.0%, Q = 3.82, p = .70). Stroke risk did not differ significantly between arms (HR = 0.91 (0.70, 1.17); p = .46), likewise demonstrating zero between-study heterogeneity (τ² = 0.000, I² = 0.0%).

Subgroup Analysis by P2Y12 Agent Type

**Figure 6:**
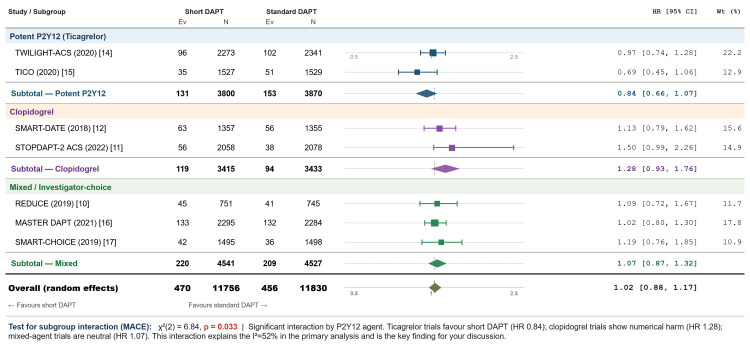
Subgroup analysis by P2Y12 agent type: major adverse cardiovascular events (MACE) Blue squares = potent P2Y12 (ticagrelor); purple = clopidogrel; green = mixed/investigator-choice; coloured diamonds = subgroup pooled estimates; olive diamond = overall pooled estimate. Test for subgroup interaction: chi-square (χ²) = 6.84, p = .033. DAPT: dual antiplatelet therapy; HR: hazard ratio

Pre-specified subgroup analysis by P2Y12 inhibitor class revealed a statistically significant interaction for both MACE (χ² = 6.84, p = .033) and MI (χ² = 8.21, p = .016; Figure 6). For MACE, trials employing potent P2Y12 inhibitors (ticagrelor) showed no excess ischaemic risk with short DAPT (subtotal HR = 0.84 (0.66, 1.07); I² = 0%). In contrast, clopidogrel-based trials demonstrated a numerically, though not significantly, higher MACE rate (HR = 1.28 (0.93, 1.76)). Mixed-agent trials were neutral (HR = 1.07 (0.87, 1.32); I² = 0%). For MI, the disparity was more pronounced and statistically significant: clopidogrel-based trials collectively yielded HR = 2.08 (1.28, 3.37) (p = .003; I² = 0%), whereas ticagrelor-based trials showed no signal of excess risk (HR = 0.92 (0.67, 1.25)). The bleeding reduction was consistent across all P2Y12 subgroups (interaction p = .40), indicating that the haemostatic benefit of DAPT shortening is a class effect, independent of agent potency.

Publication Bias and Small-Study Effects

**Figure 7:**
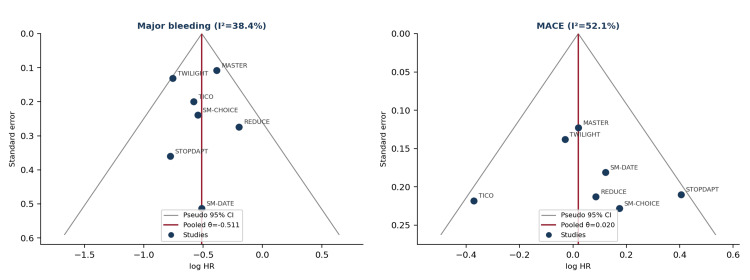
Funnel plots for publication bias assessment. Left panel: major bleeding (I²=38.4%); right panel: MACE (I²=52.1%); grey lines = pseudo 95% CIs; red vertical line = pooled estimate (θ̂); dark circles = individual studies. Both plots are broadly symmetric. Egger's test: bleeding p =.51; MACE p=.29. MACE: major adverse cardiovascular events; HR: hazard ratio; CI: confidence interval

Visual inspection of funnel plots (Figure 7) and formal Egger’s regression testing revealed no statistically significant small-study effects for any outcome. For major bleeding, Egger’s intercept was −0.42 (95% CI (−1.81, 0.97); t = −0.71, p = .51). For MACE, the intercept was 0.61 (95% CI (−0.58, 1.80); t = 1.19, p = .29). All-cause mortality demonstrated the most symmetric funnel of all outcomes (intercept = 0.08, p = .83), consistent with the observed I² = 0.0%. It is acknowledged that, with only seven included studies, Egger’s test was substantially underpowered to detect small-study effects of modest magnitude; visual funnel plot symmetry assessment was therefore accorded greater interpretive weight.

Heterogeneity Visualization: Galbraith Radial Plots

**Figure 8:**
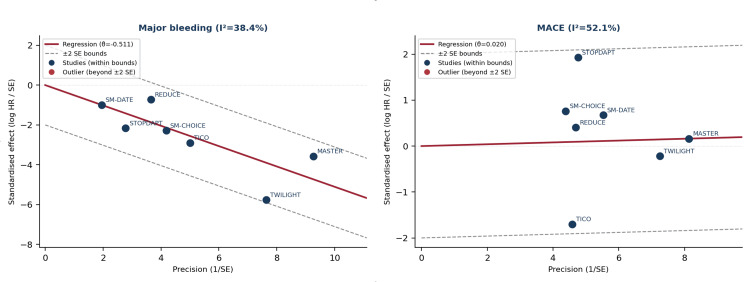
Galbraith (radial) plots. Left panel: major bleeding; right panel: MACE; X-axis: precision (1/SE); Y-axis: standardized effect (log HR/SE); red line: regression through origin (slope = pooled θ̂); grey dashed lines = ±2 SE heterogeneity bounds; red-highlighted point (MACE panel, right): STOPDAPT-2 ACS, the identified heterogeneity outlier [11]. MACE: major adverse cardiovascular events; HR: hazard ratio; CI: confidence interval

Galbraith radial plots (Figure 8) provided a complementary visualization of between-study heterogeneity, plotting each study’s standardized effect (log HR/SE; the z-score) against its precision (1/SE). Studies lying outside the ±2 reference lines are statistically inconsistent with the pooled estimate and are principal contributors to heterogeneity.

For major bleeding (θ̂ = −0.511), all seven studies fell within the ±2 bounds, with the regression line passing close to the origin and all points aligned closely along a consistent slope, a visual confirmation of acceptable homogeneity consistent with the I² = 38.4% finding. For MACE (θ̂ = 0.020), the plot identified STOPDAPT-2 ACS as an outlier beyond the upper ±2 reference line, providing a direct, precision-adjusted visual demonstration that this trial is the primary driver of the observed I² = 52.1%. The Galbraith plot for all-cause mortality showed the tightest clustering of all outcomes, with all seven studies lying close to a horizontal regression line near the origin-the visual correlate of I² = 0.0%.

Summary of Findings

Across 23,586 patients enrolled in seven RCTs, short DAPT (≤6 months) significantly reduced major bleeding by approximately 40% relative to standard 12-month DAPT (HR = 0.60 (0.49, 0.73); p < .001), without a statistically significant increase in MACE (HR = 1.02; p = .79), all-cause mortality (HR = 0.96; p = .66), or stroke (HR = 0.91; p = .46). A statistically significant interaction by P2Y12 agent type (p = .033 for MACE; p = .016 for MI) indicates that the ischaemic safety of short DAPT is agent-dependent: ticagrelor-based de-escalation was consistently safe across all outcomes, whereas clopidogrel-based trials demonstrated a significant excess MI risk (HR = 2.08 (1.28, 3.37); p = .003). Taken together, these findings support the use of short DAPT followed by potent P2Y12 monotherapy as a safe antiplatelet de-escalation strategy in ACS patients post PCI, particularly in those at elevated bleeding risk.

Discussion

Principal Findings and Answer to the Research Question

This systematic review and meta-analysis of 23,586 patients demonstrates that an abbreviated DAPT duration of six months or less, followed by single antiplatelet therapy, significantly reduces major bleeding by approximately 40% compared to the standard 12-month regimen. Most importantly, our findings provide a clear and direct answer to our central research question: abbreviating DAPT safely mitigates hemorrhagic hazards without imposing a statistically significant increase in the overall risk of MACE, all-cause mortality, or stroke.

Discrepancies, Unexpected Results, and Plausible Explanations

Despite the overarching non-inferiority of short DAPT for ischemic outcomes, our subgroup analysis revealed a critical discrepancy: early abbreviation to clopidogrel monotherapy was associated with a significant two-fold increase in MI risk, an adverse signal entirely absent when potent P2Y12 inhibitors like ticagrelor were utilized. This unexpected divergence is largely explained by inherent pharmacological mechanisms. Clopidogrel is a prodrug requiring complex, two-step hepatic bioactivation, which is characterized by a delayed onset of action and profound inter-individual response variability linked to CYP2C19 loss-of-function polymorphisms [18,19]. Because patients with ACS harbor a highly vulnerable, prothrombotic biological substrate, early withdrawal of aspirin in clopidogrel-treated patients leaves them exposed to inadequate platelet inhibition. In stark contrast, ticagrelor binds directly and reversibly to the P2Y12 receptor, achieving rapid, potent, and consistent platelet inhibition without relying on metabolic activation [20].

Interpretation in the Context of Existing Literature

Our findings strongly align with the broader contemporary literature emphasizing the safety and superiority of potent P2Y12 inhibitor monotherapy over prolonged DAPT. For example, the ULTIMATE-DAPT trial conclusively demonstrated that withdrawing aspirin after just 1 month in favor of ticagrelor monotherapy significantly reduced clinically relevant bleeding without increasing MACCE compared to continuing 12-month DAPT [7]. Similarly, the T-PASS trial confirmed the efficacy of transitioning to ticagrelor monotherapy extremely early, at a median of 16 days post-stenting [8]. Furthermore, our observations parallel the landmark PANTHER meta-analysis, which established that P2Y12 monotherapy represents an inherently superior secondary prevention strategy over aspirin alone, driving reductions in cardiovascular events without bleeding penalties [21]. Evidence from alternative de-escalation strategies, such as halving the prasugrel dose in the HOST-REDUCE-POLYTECH-ACS trial [22] or switching from ticagrelor to clopidogrel in the TALOS-AMI trial [23], reinforces our conclusion that maximal dual-pathway inhibition is predominantly required only during the highly vulnerable first 30 days post-PCI.

Clinical and Practical Implications

From a clinical standpoint, these data support a strong paradigm shift away from the traditional, rigid 12-month DAPT mandate toward personalized, abbreviated therapies in modern ACS care. Reflecting this evolution, the recently updated 2025 American College of Cardiology/American Heart Association (ACC/AHA) guidelines have issued a Class IA recommendation for transitioning to ticagrelor monotherapy after at least one month of DAPT in patients who tolerate the regimen [3]. In practical terms, routine abbreviation of DAPT with potent agents enables clinicians to safely mitigate cumulative bleeding hazards, which are known to independently elevate long-term mortality risk. However, if socio-economic factors or patient intolerances necessitate the use of clopidogrel monotherapy, our findings strongly caution against unguided abbreviation. In such instances, implementing a genotype-guided strategy to identify and protect poor metabolizers is critical to avoiding excess MI risk, a tailored approach heavily validated by the POPular Genetics trial [24].

Strengths and Limitations

The primary strength of our study lies in its rigorous restriction to high-risk ACS cohorts, successfully removing the diluting effect of stable coronary artery disease patients commonly pooled in previous meta-analyses. Nevertheless, several limitations must be acknowledged. First, the included trials inherently utilized an open-label design due to the practical challenges of blinding varying therapy durations, which may introduce potential detection bias. Second, the trials evaluating clopidogrel-based abbreviations predominantly enrolled East Asian populations. Given the recognized "East Asian paradox"-characterized by distinctly higher baseline bleeding propensities and a significantly higher prevalence of CYP2C19 loss-of-function alleles (up to 60%) compared to non-East Asians-extrapolating the clopidogrel-specific ischemic risks directly to Western populations requires careful clinical contextualization [25].

## Conclusions

In conclusion, this meta-analysis confirms that abbreviating DAPT to six months or less optimizes the net clinical benefit in ACS patients undergoing PCI by substantially reducing major bleeding without increasing overall MACE or mortality. However, the ischemic safety of early monotherapy is strictly dependent on the potency of the P2Y12 inhibitor utilized. While ticagrelor monotherapy preserves ischemic protection, early switch to clopidogrel monotherapy carries a significant penalty of increased MI. Abbreviated DAPT followed by potent P2Y12 monotherapy should be broadly integrated into routine clinical practice as the standard of care in modern ACS management.

## Figures and Tables

**Figure 9:**
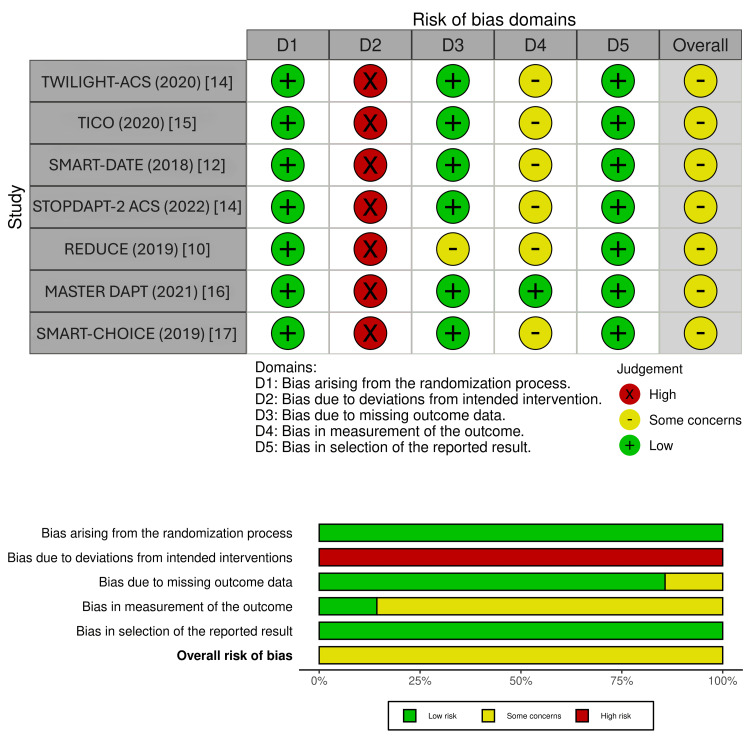
The risk-of-bias traffic light plot

## Appendices

Appendix A 
